# Major life event – diagnosis of schizophrenia in offspring and risk for cancer

**DOI:** 10.1038/sj.bjc.6601695

**Published:** 2004-03-02

**Authors:** S O Dalton, T M Laursen, P B Mortensen, C Johansen

**Affiliations:** 1 Danish Cancer Society, The Institute of Cancer Epidemiology, Strandboulevarden 49, DK-2100 Copenhagen, Denmark; 2 National Centre for Register-based Research, Aarhus University, Aarhus, Denmark

**Keywords:** neoplasms, stress, psychological, life change events, cohort studies, registries

## Abstract

The overall risk for cancer among 19 856 parents of schizophrenic patients in Denmark was not increased; however, a 30% increased risk for lung cancer was observed in mothers (95% confidence interval, 1.03–1.61), suggesting an indirect effect on cancer risk mediated by life-style factors.

The belief that psychological stress causes cancer has a long history, but scientific evidence remains contradictory to date (Fox, 1995; Dalton *et al*, 2002). Psychological stress has effects on the endocrine and immune systems, which might enhance neoplastic growth by increasing the levels of corticosterone, prolactin or estrogen, or by decreasing the number and activity of lymphocytes and natural killer cells (Ader *et al*, 1995; Kiecolt-Glaser *et al*, 2002). Stress could also increase adverse life-style behaviours like smoking and heavy alcohol drinking, which over time increase cancer incidence (Smith and Gallo, 2000).

A son or daughter with severe mental illness, such as schizophrenia, is associated with significant grief, sorrow and feeling of loss constituting psychological distress among family members (Czuchta and McCay, 2001; Martens and Addington, 2001). We investigated the effect of the diagnosis of schizophrenia in a son or daughter as an indicator of psychological stress on overall and specific cancer incidence among their parents in a population-based study based on nationwide registers with complete and unbiased information on both exposure and outcomes of interest.

## MATERIALS AND METHODS

From the nationwide Danish Central Population Register (CPR) we identified all 2 013 848 parents born in Denmark, alive on 1 April 1969 whose children were born after 1935 and were living between 1 April 1969 and 1 January 1983. Only parents whose children were over the age of 15 years at the termination of follow-up on 31 December 1997 were included since less than 1.5% of schizophrenia cases develop before that age (Westergaard *et al*, 1999). The CPR was established in 1968 and all residents of Denmark were assigned a unique identification number that permits accurate linkage between registries.

By linkage to the nationwide Danish Psychiatric Central Register, which contains information on all psychiatric admissions since 1969 (Munk-Joergensen and Mortensen, 1997), we identified and excluded 6676 parents who had been admitted for schizophrenia according to the International Classification of Diseases 8th revision between 1969 and 1993 (ICD-8, 295) and the 10th revision between 1993 and 1997 (ICD-10, F20; F25). Further, 10 466 offspring of the remaining parents were identified in the Danish Psychiatric Central Register with a diagnosis of schizophrenia; their parents constituted the exposed cohort.

We used the nationwide Danish Cancer Registry, which contains information on all cancer diagnosed in Denmark since 1943 coded according to a modified Danish version of the ICD-7 (Storm *et al*, 1997), to identify parents who developed a first primary cancer between 1 April 1969 or the day when the offspring reached 15 years (study entry), whichever date came last, and the date of a diagnosis of any cancer except nonmelanoma skin cancer, death, emigration or 31 December 1997, whichever date came first. Any diagnosis of nonmelanoma skin cancer was identified and counted, but did not lead to termination of follow-up, and a cancer occurring subsequently was counted as a first primary cancer.

The outcomes of interest were all first primary incident cancers, breast cancer, lung cancer and four subgroups of cancer hypothesised *a priori* to be associated with psychological stress: smoking-related cancers, alcohol-related cancers, virus- and immune-related cancers and hormone-related cancers.

The relative risk (RR) among parents with schizophrenic offspring relative to parents without schizophrenic offspring was estimated by log-linear Poisson regression with the GENMOD procedure in SAS 8.01. The RRs were calculated by sex, adjusted for age and calendar period (in 5-year categories) and number of children. All covariates were treated as time-dependent variables using the categories with the largest number of cases as the references. The parents were classified as exposed only from the date of schizophrenia diagnosis in the offspring and thus, parents contributed person-years at risk to the nonexposed group until schizophrenia is diagnosed in the offspring. From that date onwards the parents were contributing person-years as exposed.

## RESULTS

There were 19 856 parents in the exposed cohort and 1 979 216 parents in the unexposed cohort. The 1 999 072 cohort members had an average follow-up time of 24.2 years and contributed 48 343 429 person-years at risk.

The overall risk for cancer among parents whose offspring were diagnosed with schizophrenia was 1.00 (95% CI, 0.92–1.07) for fathers and 0.97 (95% CI, 0.89–1.05) for mothers (Table 1

**Table 1:** Relative risk of cancer overall and some groups of cancers in parents with schizophrenic offspring

	**Fathers**						**Mothers**					
	**Exposed**		**Unexposed**				**Exposed**		**Unexposed**			
	**Cases**	**Person-years**	**Cases**	**Person-years**	**RR**	**95% CI**	**Cases**	**Person-years**	**Cases**	**Person-years**	**RR**	**95% CI**
All cancers	662	60 069	104 685	23 366 679	1.00	0.92–1.07	602	70 992	105 732	24 845 689	0.97	0.89–1.05
												
Smoking-related cancers	242	61 396	39 615	23 609 014	0.94	0.82–1.06	127	74 506	15 375	25 430 296	1.14	0.95–1.35
Lung cancer	102	62 205	17 583	23 727 274	0.87	0.71–1.05	76	74 769	7934	25 464 925	1.30	1.03–1.61
Other smoking related	140	61 526	22 032	23 630 858	1.00	0.84–1.17	51	74 600	7441	25 439 990	0.97	0.71–1.26
												
Alcohol-related cancers	51	62 110	6899	23 725 922	1.21	0.90–1.57	13	74 828	2680	25 466 338	0.69	0.38–1.14
												
Virus- and immune-related cancers	160	62 119	24 033	23 689 252	1.06	0.90–1.23	124	74 259	25 795	25 341 032	0.87	0.73–1.04
												
Hormone-related cancers	50	61 890	9404	23 689 252	0.77	0.58–1.01	241	72 661	42 055	25 169 415	1.04	0.92–1.18
Breast cancer	1	62 333	164	23 748 183	—*	—	154	73 328	30 261	25 248 345	0.98	0.84–1.15

RR = relative risk adjusted for age, calendar period and number of children; smoking-related cancers; cancers of the buccal cavity (ICD-7 140–148), oesophagus (ICD-7 150), pancreas (ICD-7 157), larynx (ICD-7 161), lung (ICD-7 162), urinary bladder (ICD-7 180); kidney (ICD-7 181); alcohol-related cancers, cancers of the tongue (ICD-7 141), mouth (ICD-7 143–144), pharynx (ICD-7 145–148), oesophagus (ICD-7 150), liver (ICD-7 155), larynx (ICD-7 161); virus- and immune-related cancers, cancers of the liver (ICD-7 155), cervix uteri (ICD-7 171), nonmelanoma skin (ICD-7 191), non-Hodgkin's lymphoma (ICD-7 200, 202), leukaemia (ICD-7 204); hormone-related cancers, cancers of the breast (ICD-7 170), uterus (ICD-7 172), ovary (ICD-7 175) and prostate (ICD-7 177);

* No RR calculated for breast cancer in fathers due to too few cases.

). For mothers the increased risk for all smoking-related cancers was carried by an increased risk for lung cancer of 1.30 (95% CI, 1.03–1.61) whereas the estimate for other smoking-related cancers combined was 0.97 (95% CI, 0.72–1.26). In fathers the risk for lung cancer was 0.87 (95% CI, 0.71–1.05) and for other smoking-related cancers combined was 1.00 (95% CI, 0.84–1.17). The maternal RR for breast cancer was 0.98 (95% CI, 0.84–1.15) and there was no excess risk for any of the groups of alcohol-, virus- and immune- or hormone-related cancers in either mothers or fathers.

## DISCUSSION

We found no evidence that schizophrenia in an offspring was associated with an increased risk of overall cancer in the parents. There was an excess risk for lung cancer in mothers to schizophrenic offspring but this was not observed in fathers.

Our study has the advantage of access to nationwide registries with complete coverage (Munk-Joergensen and Mortensen, 1997; Storm *et al*, 1997) which essentially eliminates recall and selection bias, loss to follow-up or misclassification of exposures or outcomes. We expected the chosen exposure would stress all parents, regardless of personality, coping style and social support or network. If there is a threshold effect for stress on cancer risk, we believe that the exposure is a strain of such level and duration that most of the exposed cohort members would be considered above this threshold.

Our study is in line with previous studies, suggesting that increased overall cancer risk attributable to stressful life events is small if any at all (McGee *et al*, 1996; Dalton *et al*, 2002). Further, the only association with cancer was observed in mothers of offspring with schizophrenia, who had an elevated risk for lung cancer. This finding may reflect a stress-induced increase in the amount smoked (Anda *et al*, 1990).

Taken together the evidence from this analysis provides no support for a direct effect of psychological stress on cancer aetiology.
